# Dr. C. C. P. Clark

**Published:** 1899-02

**Authors:** 


Dr. C. C. P. Clark, of Oswego, N. Y., died at his residence in that, city Wednesday, January 11, 1899, aged 76 years. He was one of the most prominent physicians in that region, and a man of wide learning. He was born at Middlebury, Vt., graduated at Middlebury College in 1843 and took his doctorate degree at the College of Physicians and Surgeons, New York, in 1847.

